# P-1380. Comparative Study Of Fixed-Dose Combinations Versus Separate Tablets In The Treatment Of Extrapulmonary Tuberculosis

**DOI:** 10.1093/ofid/ofaf695.1567

**Published:** 2026-01-11

**Authors:** Fatma Hammami, Khaoula Rekik, Amal Chakroun, Makram Koubaa, Fatma Smaoui, Chakib Marrakchi, Mounir Ben Jemaa

**Affiliations:** Infectious Diseases Department, Hedi Chaker University Hospital, University of Sfax, Tunisia, Sfax, Sfax, Tunisia; Infectious Diseases Department, Hedi Chaker University Hospital, University of Sfax, Tunisia, Sfax, Sfax, Tunisia; Infectious Diseases Department, Hedi Chaker University Hospital, University of Sfax, Tunisia, Sfax, Sfax, Tunisia; Infectious Diseases Department, Hedi Chaker University Hospital, University of Sfax, Tunisia, Sfax, Sfax, Tunisia; Infectious Diseases Department, Hedi Chaker University Hospital, University of Sfax, Tunisia, Sfax, Sfax, Tunisia; Infectious Diseases Department, Hedi Chaker University Hospital, University of Sfax, Tunisia, Sfax, Sfax, Tunisia; Infectious Diseases Department, Hedi Chaker University Hospital, University of Sfax, Tunisia, Sfax, Sfax, Tunisia

## Abstract

**Background:**

Tuberculosis remains a major global public health challenge. To minimize treatment failure and the emergence of drug resistance, the World Health Organization recommends the use of fixed-dose combinations (FDCs) for antituberculosis therapy. This study aimed to study the efficacy and tolerability of FDCs in comparison with separate tablets in the management of extrapulmonary tuberculosis.

**Methods:**

We conducted a retrospective study including all patients hospitalized for extrapulmonary tuberculosis in the infectious diseases department between 2000 and 2024. Patients were treated with either FDCs or separate tablets.

**Results:**

We encountered 562 cases, among whom 358 (63.7%) were women. The mean age was 40 ± 19 years. In total, 241 patients (42.9%) received FDCs and 321 patients (57.1%) received separate tablets. Gastrointestinal adverse events, including vomiting (5.3% vs 0.4%; p = 0.001) and abdominal pain (6.5% vs 2.5%; p = 0.026), as well as neurological symptoms (14.3% vs 3.7%; p < 0.001), were significantly more frequent among patients receiving separate tablets. No significant differences were observed between the groups regarding cutaneous reactions (6.2% vs 8.7%; p = 0.26), hematological abnormalities (2.8% vs 4.5%; p = 0.29), or hepatic cytolysis (14.6% vs 17.4%; p = 0.37). Clinical outcomes were more favorable in the FDC group, with lower rates of complications (13.3% vs 25.9%; p < 0.001), sequelae (9.5% vs 16.5%; p = 0.017), and relapse (2.1% vs 5.9%; p = 0.031). Cure rates (92.2% vs 90%; p = 0.36) and mortality (2.8% vs 1.2%; p = 0.21) were comparable between the two groups.

**Conclusion:**

Treatment of extrapulmonary tuberculosis with FDCs was associated with less neurological and gastrointestinal adverse events and improved clinical outcomes compared to separate tablet therapy. These findings support the preferential use of FDCs to optimize treatment tolerability and reduce relapse rates.

**Disclosures:**

All Authors: No reported disclosures

